# Radiolabeled Probes Targeting Hypoxia-Inducible Factor-1-Active Tumor Microenvironments

**DOI:** 10.1155/2014/165461

**Published:** 2014-08-18

**Authors:** Masashi Ueda, Hideo Saji

**Affiliations:** ^1^Department of Pharmaceutical Analytical Chemistry, Graduate School of Medicine, Dentistry, and Pharmaceutical Sciences, Okayama University, 1-1-1 Tsushima-naka, Kita-ku, Okayama 700-8530, Japan; ^2^Department of Patho-Functional Bioanalysis, Graduate School of Pharmaceutical Sciences, Kyoto University, Kyoto 606-8501, Japan

## Abstract

Because tumor cells grow rapidly and randomly, hypoxic regions arise from the lack of oxygen supply in solid tumors. Hypoxic regions in tumors are known to be resistant to chemotherapy and radiotherapy. Hypoxia-inducible factor-1 (HIF-1) expressed in hypoxic regions regulates the expression of genes related to tumor growth, angiogenesis, metastasis, and therapy resistance. Thus, imaging of HIF-1-active regions in tumors is of great interest. HIF-1 activity is regulated by the expression and degradation of its *α* subunit (HIF-1*α*), which is degraded in the proteasome under normoxic conditions, but escapes degradation under hypoxic conditions, allowing it to activate transcription of HIF-1-target genes. Therefore, to image HIF-1-active regions, HIF-1-dependent reporter systems and injectable probes that are degraded in a manner similar to HIF-1*α* have been recently developed and used in preclinical studies. However, no probe currently used in clinical practice directly assesses HIF-1 activity. Whether the accumulation of ^18^F-FDG or ^18^F-FMISO can be utilized as an index of HIF-1 activity has been investigated in clinical studies. In this review, the current status of HIF-1 imaging in preclinical and clinical studies is discussed.

## 1. Introduction

Insufficient blood supply to a rapidly growing solid tumor leads to hypoxia—oxygen tension that is below physiological levels. The median value of partial oxygen pressure (pO_2_) is reportedly 10 mmHg in breast cancers but is 65 mmHg in normal breast tissue [1]. Hypoxic regions are not only critically important in tumor physiology and treatment, but also strongly associated with malignant progression, therapeutic resistance, and poor prognosis [2–4]. In such regions, the transcription factor hypoxia-inducible factor-1 (HIF-1) is overexpressed. HIF-1, one of the critical components of hypoxic responses, is a master transcriptional activator of various genes related to malignant tumor phenotypes [5–7]. Therefore, the development of techniques to noninvasively detect HIF-1-active hypoxic tumor cells has received considerable interest. Such techniques will provide useful information that can be applied to qualitative diagnosis of tumors and development of therapeutic strategies for cancer.

## 2. HIF-1 Biology

**Figure 1:**
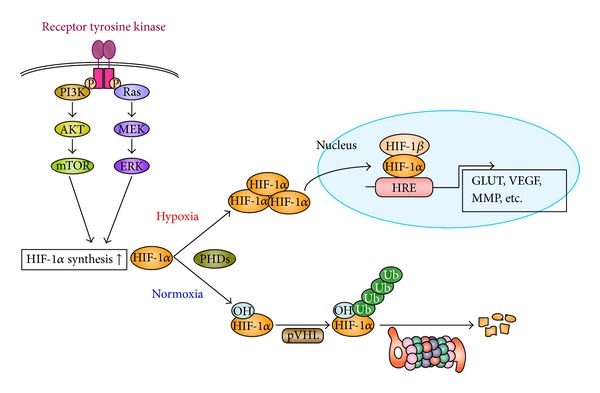
Regulatory mechanism of hypoxia-inducible factor (HIF-1) expression. Growth factor stimulation induces HIF-1*α* subunit (HIF-1*α*) protein synthesis by activating PI3K/Akt/mTOR and Ras/MEK/ERK kinase pathways. Under normoxic conditions, HIF-1*α* is hydroxylated by proline hydroxylases (PHDs), triggering its interaction between von Hippel-Lindau tumor suppressor protein (pVHL), leading to its polyubiquitination and subsequent proteasomal degradation. In contrast, under hypoxic conditions, HIF-1*α* remains stable, enters the nucleus, and, together with HIF-1*β*, binds to hypoxia-responsive elements (HREs), upregulating the expression of target genes such as glucose transporters (GLUTs), vascular endothelial growth factors (VEGFs), and matrix metalloproteinases (MMPs). Ub: ubiquitin.

HIF-1 is a heterodimeric transcription factor that consists of *α* and *β* subunits (HIF-1*α* and HIF-1*β*, resp.) [8]. HIF-1*β* is a constitutively expressed nuclear protein; however, HIF-1*α* expression is regulated at both the translational and posttranslational levels. Growth signaling through receptor tyrosine kinases activates PI3K/Akt/mTOR and Ras/MEK/ERK signaling pathways and increases HIF-1*α* translation (Figure 1) [9–11]. In contrast, HIF-1*α* expression is maintained at low levels in most normoxic tissues via a posttranslational regulation mechanism—oxygen-dependent proteasomal degradation. Under normoxic conditions, Pro^402^ and Pro^564^ in the HIF-1*α* oxygen-dependent degradation domain (ODD) are hydroxylated by proline hydroxylases [12–14]. The von Hippel-Lindau tumor suppressor protein (pVHL) specifically recognizes these hydroxylated prolines and interacts with HIF-1*α*. pVHL forms a complex with Elongin BC, Cul2, and Rbx1 [15], and this complex, which acts as a ubiquitin ligase, polyubiquitinates HIF-1*α*, leading to its degradation in the 26S proteasome [16, 17]. However, proline residue hydroxylation does not occur under hypoxic conditions, allowing HIF-1*α* to escape degradation and move into the nucleus where it associates with HIF-1*β* and exerts its transcriptional activity by binding to hypoxia-responsive elements (HREs) (Figure 1) [18, 19]. Because HIF-1*β* is constitutively expressed in the nucleus, the transcriptional activity of HIF-1 is regulated by HIF-1*α* expression and degradation.

HIF-1*α* expression is reported to increase dramatically in conditions where pO_2_ is less than 6% (40 mmHg) [20]. However, the pO_2_ threshold values that lead to HIF-1*α* stabilization vary among different organs [21]. Because pulmonary cells are consistently exposed to relatively high pO_2_, the values that these cells perceive as abnormal are relatively high [11]. In contrast, the bone marrow is the only tissue that expresses HIF-1*α* protein under normal physiological conditions [22]. Therefore, HIF-1*α* is stably present in “biologically hypoxic regions,” where the tissues and/or cells themselves sense oxygen deficiency. These regions do not coincide completely with “physically hypoxic regions,” where pO_2_ is less than 10 mmHg. Thus, HIF-1-dependent probes or reporter systems are required to directly visualize HIF-1-active hypoxic regions.

## 3. Monitoring HIF-1 Activity* In Vivo* by Using HIF-1-Dependent Reporter Gene Imaging

For the first time, nuclear medical molecular imaging of HIF-1 activity* in vivo* has been performed using a HIF-1-dependent reporter system. In the field of molecular biology, reporter systems are commonly used to monitor the expression of a gene of interest. To directly monitor HIF-1 activity, cell lines that stably contain a reporter gene suitable for* in vivo* imaging downstream of HRE repeats have been established. When implanted into animals, these cells express the reporter protein in a HIF-1-dependent manner, and the amount of reporter protein expression can be measured using a molecular probe that binds to or is metabolized by these proteins.

Serganova et al. [23] and Wen et al. [24] performed positron emission tomography (PET) imaging to detect HIF-1 activity in tumors, using herpes simplex virus type 1 thymidine kinase (HSV1-TK) as a reporter gene; a PET reporter of* in vivo* gene expression has been widely used [25, 26]. Although endogenous TKs are normally present in mammalian cells, their substrate specificity differs from that of HSV-1TK. Because HSV1-TK has broader specificity than mammalian TKs, a probe that is selectively phosphorylated by viral TKs can be used to visualize HSV1-TK expression [27]. Serganova et al. [23] constructed a retroviral vector bearing the HRE-HSV1-TK reporter gene and transfected it into C6 glioma cells. The reporter system showed that dose-dependent patterns in the temporal dynamics of HIF-1 transcriptional activity were induced by decreased pO_2_. PET imaging using 2′-[^18^F]fluoro-2′-deoxy-1*β*-d-arabinofuranosyl-5-ethyl-uracil (^18^F-FEAU), a radioactive probe selectively metabolized and retained in HSV1-TK-expressing cells, clearly visualized tumors. The spatial heterogeneity of HIF-1 transcriptional activity as a function of tumor size was shown in a study of reporter xenografts in mice. With increasing tumor diameter (>3 mm), HIF-1 transcriptional activity markedly increased in the tumor core regions. This marked one of the earliest successes in nuclear medical molecular imaging for HIF-1 activity* in vivo*. Since then, several approaches for noninvasive imaging of HIF-1 activity have been reported, including those using different combinations of reporter genes and radiolabeled probes such as sodium-iodide symporter and ^99m^Tc-pertechnetate [28], glycosylphosphatidylinositol-anchored avidin and ^67^Ga-DOTA-biotin [29], and HSV1-TK and 9-(4-[^18^F]fluoro-3-hydroxymethyl-butyl)guanine (^18^F-FHBG) [30]. Brader et al. [31] constructed RH7777 Morris hepatoma cells bearing a triple reporter gene (HRE-HSV1-TK/green fluorescent protein/firefly luciferase) and prepared an orthotopic liver tumor model. They performed ^18^F-FEAU-PET and luciferase bioluminescence imaging and succeeded in hypoxia-driven reporter imaging using both techniques.

HIF-1-dependent reporter systems are also useful for comparing HIF-1 activity and exogenous markers of tumor hypoxia. Wen et al. [24] generated rat prostate adenocarcinoma cells containing HRE-HSV1-TK fused to enhanced green fluorescent protein as a reporter. PET imaging was performed using 2′-fluoro-2′-deoxy-1*β*-d-arabinofuranosyl-5-[^124^I]iodouracil (^124^I-FIAU), and the distributions of ^124^I-FIAU and the exogenous hypoxic cell marker [^18^F]fluoromisonidazole (^18^F-FMISO) were compared. ^124^I-FIAU PET imaging of hypoxia-induced reporter gene expression was feasible, and the intratumoral distributions of ^124^I-FIAU and ^18^F-FMISO were similar. Similar results were obtained for human colorectal HT29 cancer cells bearing the same reporter system [32]. However, no significant correlation between the ^18^F-FMISO PET and HIF-1-dependent reporter readouts has also been reported [33]. The reasons for the discrepancy between these findings remain unclear and warrant further investigation. Notably, these described reporter systems did not include the ODD in the expressed reporter proteins. The degradation of HIF-1*α* is reported to occur within a few minutes under normoxic conditions [34]. Thus, although reporter protein expression was regulated by HIF-1, it is necessary to acknowledge that the reporter proteins may not necessarily reflect real-time HIF-1 activity once the expression was completed.

## 4. Molecular Imaging of HIF-1-Active Tumor Microenvironments by Using Probes with Oxygen-Dependent Degradation

HIF-1-dependent reporter imaging systems are excellent tools not only for obtaining spatiotemporal information regarding HIF-1 expression, but also for evaluating the effectiveness of hypoxia-targeting therapies* in vivo*. However, these systems require exogenous gene transfection and are difficult to apply to humans. To overcome this limitation, a number of injectable probes containing ODD that degrade in an oxygen-dependent manner have been developed [35–41].

### 4.1. Chimeric Fusion Protein Probes Containing Oxygen-Dependent Degradation Domains

**Figure 2:**
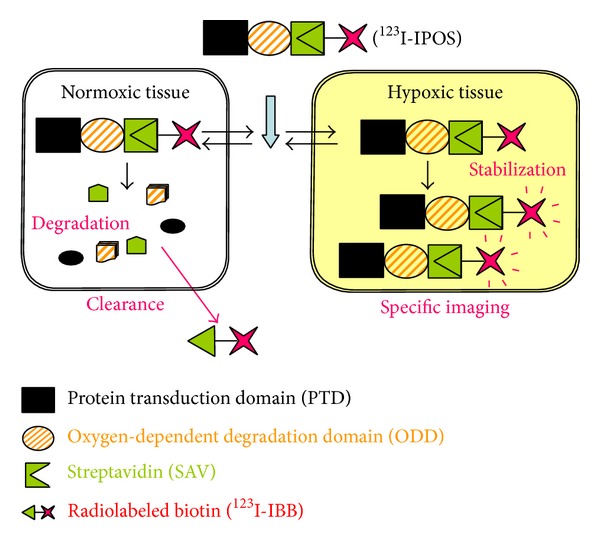
Principle underlying the imaging of HIF-1-active tumor microenvironments, using ^123^I -IPOS. The PTD enables ^123^I-IPOS to be delivered to all tissues. In normoxic tissues, oxygen-dependent POS degradation occurs and ^123^I-IBB loses its binding partner and is cleared. In contrast, in hypoxic tissues, ^123^I-IPOS escapes degradation and is retained within cells owing to its molecular size. Thus, ^123^I-IPOS allows for specific imaging of HIF-1-active hypoxic microenvironments.

To design a probe that degraded in a manner similar to HIF-1*α*, Kudo et al. [35] selected HIF-1*α*_548–603_, the region of ODD that is essential for oxygen-dependent degradation [42], as the core structure of the probe. An amino acid sequence that increases cell membrane permeability—the protein transduction domain (PTD) [43]—was included in the probe to facilitate its transport into cells. PTD-ODD was fused to monomeric streptavidin (SAV), generating the chimeric protein PTD-ODD-SAV (POS). POS was then labeled with the radioiodinated biotin derivative (3-[^123^I]iodobenzoyl)norbiotinamide (^123^I-IBB) to produce ^123^I-IBB-POS (^123^I-IPOS) [35]. The principle behind the imaging of HIF-1-active hypoxic microenvironments by using ^123^I-IPOS is outlined schematically in Figure 2.

^125^I-IPOS showed more than 2-fold greater accumulation in cells incubated under hypoxic conditions (0.1% O_2_) than under normoxic conditions (20% O_2_). Size-exclusion high-performance liquid chromatography (HPLC) analysis revealed that more than 80% of intracellular radioactivity was derived from intact ^125^I-IPOS in the hypoxic cells. Reoxygenation analysis showed that the intracellular radioactivity of the reoxygenated group decreased by approximately 40%, compared to that under hypoxic conditions, and the radioactivity excreted into the medium were mostly attributable to ^125^I-IBB and other small molecules [35]. These findings suggest that ^125^I-IPOS is stable under hypoxic conditions but is degraded under normoxic conditions and that ^125^I-IBB is excreted from cells. Biodistribution analysis showed that radioactivity accumulation in tumors 24 h after ^125^I-IPOS administration was 1.4 ± 0.3% of the injected dose per gram of tissue, and the tumor-to-blood ratio, which serves as an index of favorable image contrast, was 5.1. Forty-eight hours after ^125^I-IPOS administration, radioactivity in the tumor decreased, but the tumor-to-blood ratio increased [35]. Single-photon emission computed tomography (SPECT)/X-ray computed tomography imaging with ^125^I-IPOS clearly visualized the tumors. Autoradiographic study showed that the intratumoral distribution of ^125^I-IPOS is heterogeneous and corresponds to HIF-1*α*-positive regions detected using immunostaining [38]. Moreover, a positive and significant correlation was observed between ^125^I-IPOS accumulation and HIF-1-dependent luciferase bioluminescent signals in the identical tumor [35]. These findings indicate that ^123^I-IPOS is a promising probe for imaging HIF-1-active hypoxic microenvironments in tumors.

### 4.2. Rapid Detection of HIF-1-Active Tumor Microenvironments by Using POS: A Pretargeting Approach

Although PET is used less widely than SPECT in nuclear medical molecular imaging, it allows for higher sensitivity and resolution, as well as high-quality quantitative imaging. Thus, PET imaging of HIF-1-active tumors can provide precise information for determining appropriate therapeutic strategies and predicting prognoses of patients. Fluorine-18 is one of the most widely used radionuclides for PET imaging because of its ease of production, low positron energy, and adequate half-life (110 min). However, the half-life is too short to obtain signals 24 h after administration, at which time clear images can be obtained using ^123^I-IPOS. Therefore, development of an imaging procedure that can be used to obtain high-contrast images during the early period after administration is required for successful PET imaging using ^18^F.

**Figure 3:**
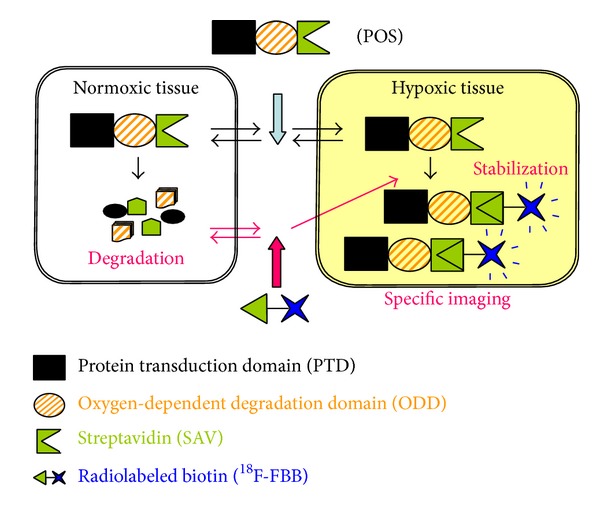
Principle underlying the imaging of HIF-1-active tumor microenvironments, using pretargeted POS and ^18^F-FBB. The PTD enables the delivery of POS to all tissues. In normoxic tissues, POS degrades in a manner similar to HIF-1*α*. In contrast, in hypoxic tissues, POS escapes degradation and is retained inside the cells. After allowing sufficient time for POS to degrade in normal tissues, ^18^F-FBB is administered. ^18^F-FBB enters cells by passive diffusion and binds to the SAV moiety of the POS retained in hypoxic cells; this does not occur in normoxic tissues. Therefore, pretargeting POS followed by ^18^F-FBB administration enables specific imaging of HIF-1-active hypoxic microenvironments.

A pretargeting approach uses a combination of tumor-targeting molecules and the prompt clearance of small radioactive compounds from the blood. One advantage of this method is that it can provide a high target: nontarget organ ratios shortly after injection. Therefore, we utilized a pretargeting approach based on the high-affinity and specific interaction between SAV and biotin for the PET imaging of HIF-1-active tumor microenvironments. The underlying principle of pretargeted imaging of HIF-1-active hypoxic microenvironments by using POS and (4-[^18^F]fluorobenzoyl)norbiotinamide (^18^F-FBB) is outlined schematically in Figure 3.

Conjugation of* N*-succinimidyl-4-[^18^F]fluorobenzoate and norbiotinamine yielded ^18^F-FBB, with a total synthesis time of 150 min, radiochemical yield of 23%, and radiochemical purity of >95%. An* in vitro* binding assay confirmed that ^18^F-FBB bound to the SAV moiety of POS. Biodistribution analysis showed that ^18^F-FBB was rapidly cleared from the body without POS pretargeting. The tumor-to-blood and tumor-to-muscle ratios were <1 at all investigated time points, indicating that ^18^F-FBB itself does not show tumor accumulation. In contrast, ^18^F-FBB accumulated in tumors in POS-pretargeted mice. ^18^F-FBB was able to enter the tumor cells by passive diffusion owing to its lipophilicity and bind to POS retained in tumor cells. Both tumor-to-blood and tumor-to-muscle radioactivity ratios increased over time [37]. Tumor-to-blood ratios comparable to those at 24 h after ^123^I-IPOS administration can be obtained within 3 h of ^18^F-FBB administration by using the pretargeting method. Pretreatment with excessive d-biotin significantly inhibited ^18^F-FBB accumulation in POS-pretargeted tumors. Size-exclusion HPLC analyses revealed that 80% of intratumoral radioactivity is attributable to macromolecules. Taken together, these findings show that ^18^F-FBB binds to POS* in vivo*, and PET imaging clearly delineated tumors within 3 h after injecting ^18^F-FBB into POS-pretargeted mice. Therefore, the pretargeting approach makes it possible to reduce the time taken from probe administration to image acquisition by 8-fold, allowing imaging to be performed within the half-life of ^18^F. In mice with tumors that had been transfected with a HIF-1-dependent luciferase reporter gene, ^18^F-FBB accumulation positively correlated with HIF-1-dependent luciferase bioluminescent signals. The ^18^F-FBB-distributed areas were consistent with HIF-1*α*-positive areas in tumors pretargeted with POS [37]. Thus, these findings demonstrate that ^18^F-FBB accumulation in the POS-pretargeted tumors reflects HIF-1-activity and the pretargeting approach with POS and ^18^F-FBB is suitable for rapid imaging of HIF-1-active tumors.

### 4.3. Peptide Probes Containing Essential Amino Acids for Oxygen-Dependent Degradation

POS is a 34 kDa protein generated in* Escherichia coli*. Although promising results for the imaging of HIF-1-active tumor microenvironments were obtained, as described above, clinical applications are limited by the possibility of immunogenicity. Peptide probes or small molecules are favorable for clinical applications because they can be easily synthesized with high purity and show no immunogenicity. Our research group succeeded in developing a peptide-based imaging probe that is degraded in a manner similar to HIF-1*α* [41].

Based on the degradation mechanism of HIF-1*α*, we selected HIF-1*α*_547–574_ as the basic structure for the oxygen-sensitive peptide probe. For site-specific radiolabeling, a glycyl cysteine was introduced into the C-terminal of the HIF-1*α*_547–574_ scaffold, yielding a peptide named OP30. OP30 was degraded when incubated with HeLa cell lysates but was unable to penetrate cell membranes. Thus, to increase its membrane permeability, the nine* N*-terminal amino acids of OP30 were replaced by l-lysine or d-lysine, producing peptides KOP30 and DKOP30, respectively. As expected, these modified peptides were also degraded in HeLa cell lysates and accumulated in the tumor cells. Degradation was inhibited by the addition of a proteasome inhibitor. Furthermore, by replacing the proline residues in KOP30 and DKOP30 that are essential for oxygen-dependent degradation with L-alanine, the resulting peptides (mKOP30 and mDKOP30, resp.,) were not degraded in HeLa cell lysates. Taken together, these results indicate that the proline residues and proteasome function are required for the degradation of KOP30 and DKOP30, similar to HIF-1*α*. Biodistribution analysis showed that ^125^I-DKOP30 had better tumor accumulation and tumor-to-blood ratio than ^125^I-KOP30. Therefore, we performed an additional study using ^123/125^I-DKOP30. Tumors were visualized clearly by planar imaging using ^123^I-DKOP30, and autoradiographic studies showed that the intratumoral distribution of ^125^I-DKOP30 was consistent with the HIF-1*α*-positive areas detected using immunohistochemical staining. Moreover, a positive and significant correlation was observed between ^125^I-DKOP30 accumulation and HIF-1-dependent luciferase bioluminescent signals within the same tumor, whereas no correlation between ^125^I-mDKOP30 accumulation and bioluminescent signals was observed. These findings indicate that ^123^I-DKOP30 is a useful peptide probe for imaging HIF-1-active tumors.

Because many peptide probes have already been used in PET/SPECT imaging in humans [44–46], ^123^I-DKOP30 has the most potential probe for imaging HIF-1-active tumors in clinical settings so far. Using planar imaging, the accumulation of ^123^I-DKOP30 in tumors was found to be higher than the background radioactivity in the cervicofacial and thoracic regions; therefore, it is possible to detect HIF-1 activity in the head and neck, breast, and lung cancers. However, the high background radioactivity in the abdominal region would hamper the imaging of HIF-1-active abdominal tumors. To overcome this issue, a probe that demonstrates greater accumulation in tumors and/or faster clearance from blood and normal abdominal organs should be developed.

## 5. Molecular Imaging of HIF-1-Active Tumor Microenvironments in Clinical Practice

No probes currently used in clinical practice can directly detect HIF-1 activity. As described in Section 2, pO_2_ values at which HIF-1*α* is stable differ between organs. Thus, in principle, probes targeting physically hypoxic regions (pO_2_ of ≤10 mmHg) are not suitable for the detection of HIF-1 expression or activity because the mechanisms underlying their hypoxic accumulation are independent of HIF-1 expression. However, HIF-1*α*-positive regions reportedly overlap with pimonidazole-adducted regions in tumors. Although microscopic observation has shown that unmatched regions also exist [47, 48], the spatial resolution of nuclear medical imaging is not sufficiently high to distinguish these unmatched regions* in vivo*. Therefore, HIF-1 activity could potentially be evaluated indirectly by using probes targeting physically hypoxic regions. In fact, a number of clinical studies have been performed recently to evaluate HIF-1 expression in areas of [^18^F]fluoromisonidazole (^18^F-FMISO) or 2-deoxy-2-[^18^F]fluoro-d-glucose (^18^F-FDG) accumulation; however, the obtained results have been discrepant. Furthermore, it should be noted that neither ^18^F-FMISO nor ^18^F-FDG can be used to evaluate directly the HIF-1 activity, even if the probe accumulation is correlated with HIF-1 activity.

### 5.1. ^18^F-FMISO and Its Derivatives

Sato et al. [49] examined the relationship between ^18^F-FMISO PET results and HIF-1*α* expression in patients with oral squamous cell carcinoma (OSCC). The maximum standardized uptake value (SUVmax) of ^18^F-FMISO PET was significantly higher in HIF-1*α*-positive patients than in those negative for HIF-1*α*. The authors concluded that ^18^F-FMISO uptake into the primary site of OSCC indicates the presence of a HIF-1*α*-positive hypoxic environment. Kawai et al. [50] evaluated the correlations between ^18^F-FMISO uptake, HIF-1*α* expression, and expression of vascular endothelial growth factor (VEGF), a HIF-1 target gene, in newly diagnosed and recurrent malignant gliomas. In contrast to the findings reported by Sato et al. [49], HIF-1*α* expression in the tumors did not correlate with ^18^F-FMISO uptake in either newly diagnosed or recurrent glioma patients. A significant but weak correlation between VEGF expression and ^18^F-FMISO uptake was observed in newly diagnosed glioma patients, but not in those with recurrent glioma. Hu et al. [51] investigated the relationship between [^18^F]fluoroerythronitroimidazole (^18^F-FETNIM) uptake and expression of HIF-1*α*, VEGF, and glucose transporter 1 (GLUT-1) in nonsmall cell lung cancer. A positive correlation existed between the tumor-to-muscle ratio of ^18^F-FETNIM and the expression of each of these HIF-1*α* targets.

### 5.2. ^18^F-FDG

Sato et al. [49] examined the relationship between the SUVmax of ^18^F-FDG and HIF-1*α* expression in OSCC, and no significant correlation was observed. This finding is in accordance with that reported by Cheng et al. [52] who found no correlation between ^18^F-FDG uptake and HIF-1*α* expression in breast cancer patients. Conversely, Takebayashi et al. [53] investigated the relationship between the SUVmax of ^18^F-FDG, HIF-1*α* expression, and GLUT-1 expression in gastric cancer patients. The SUVmax was correlated with HIF-1*α* expression, but, interestingly, not with GLUT-1 expression. In this case, ^18^F-FDG accumulation could indicate tissue hypoxia rather than glucose transport activity in aggressive cancer growths.

## 6. Conclusion

In this paper, the current status of nuclear medical molecular imaging strategies for HIF-1-active tumor microenvironments was discussed. At the preclinical level, HIF-1 activity has been directly evaluated by using HIF-1-dependent reporter systems and injectable probes that degrade in a manner similar to HIF-1*α*. However, these techniques have not yet been applied clinically. At the clinical level, whether the accumulation of ^18^F-FDG or ^18^F-FMISO can be utilized as an index of HIF-1 activity has been investigated but results obtained in these studies varied substantially. To address this issue, the development of radiolabeled probes that directly evaluate HIF-1 activity in humans is required.
